# Newly diagnosed glioblastoma: adverse socioeconomic factors correlate with delay in radiotherapy initiation and worse overall survival

**DOI:** 10.1093/jrr/rrx103

**Published:** 2018-02-08

**Authors:** Erqi L Pollom, Dylann K Fujimoto, Summer S Han, Jeremy P Harris, Suzanne A Tharin, Scott G Soltys

**Affiliations:** 1 Department of Radiation Oncology, Stanford University School of Medicine, Stanford, CA, USA; 2 Department of Medicine, Stanford University School of Medicine, Stanford, CA, USA; 3 Department of Neurosurgery, Stanford University School of Medicine, Stanford, CA, USA

**Keywords:** glioblastoma, chemoradiation, treatment timing

## Abstract

The optimal time for starting radiation in patients with glioblastoma (GBM) is controversial. We aimed to evaluate postoperative radiotherapy treatment patterns and the impact of timing of radiotherapy on survival outcomes in patients with GBM using a large, national hospital-based registry in the era of Stupp chemoradiation. We performed a retrospective cohort study using the National Cancer Data Base and identified adults with GBM diagnosed between 2010 and 2013 and treated with chemoradiation. We classified time from surgery/biopsy to radiation start into the following categories: <15 days, 15–21 days, 22–28 days, 29–35 days, 36–42 days and >42 days. We assessed the relation between time to radiation start and survival using Cox proportional hazards modeling adjusting for clinically relevant variables that were selected *a priori*. We used multivariate logistic modeling to determine factors independently associated with receipt of delayed radiation treatment. A total of 12 738 patients met our inclusion criteria after our cohort selection process. The majority of patients underwent either gross total (*n* = 5270, 41%) or subtotal (*n* = 4700, 37%) resection, while 2768 patients (22%) underwent biopsy only. Median time from definitive surgery or biopsy to initiation of radiation was 29 days (interquartile range 24–36 days). For patients who had biopsy or subtotal resection, earlier initiation of radiation did not appear to be associated with improved survival. However, among patients who underwent gross total resection, there appeared to be improved survival with early initiation of radiation. Patients who initiated radiation within 15–21 days of gross total resection had improved survival (hazard ratio 0.82, 95% confidence interval 0.69–0.98, *P* = 0.03) compared with patients who had delayed (>42 days after surgery) radiation. There was also a trend (*P* = 0.07 to 0.12) for improved survival for patients who initiated radiation within 22–35 days of gross total resection compared with patients who had delayed radiation. Patients who were black, had Medicaid or other government insurance or were not insured, and who lived in metropolitan areas or further away from the treating facility had higher odds of receiving radiation >35 days after gross total resection. Patients who lived in higher income areas had higher odds of receiving radiation within 35 days of a gross total resection. In a large cohort of patients with GBM treated with chemoradiation, our data suggest a survival benefit in initiating radiotherapy within 35 days after gross total resection. Further research is warranted to understand barriers to timely access to optimal therapy.

## INTRODUCTION

Survival outcomes are poor for patients diagnosed with glioblastoma (GBM), despite standard of care therapies, including surgery, radiotherapy and temolozomide. There has been continued effort to optimize the delivery of radiotherapy in these patients in order to improve outcomes. For many cancers, including breast and head and neck histologies, delay in initiation of radiotherapy has been associated with worse local control and survival outcomes [1]. Patients with rapidly growing tumors such as GBMs are potentially most at risk of the negative consequences of treatment delay [2].

However, the optimal time to start radiation in patients with GBM is controversial. Although some institutional, retrospective series have shown an adverse impact on survival with radiation delay after surgery, others have shown no impact [3–7]. A secondary analysis of the Radiation Therapy Oncology Group database of patients with glioblastoma showed no evidence of worse survival in delaying initiation of radiation for up to 6 weeks and, interestingly, superior survival in patients for whom radiation was delayed beyond 4 weeks from surgery [3]. Selection bias may explain these retrospective findings of survival detriment with early initiation of radiation: patients with the most adverse characteristics are likely the ones for whom physicians started radiation sooner. Additionally, small numbers of patients and heterogeneous, outdated therapies used in many of these studies make interpretation of the results challenging.

To address the limitations of these prior studies, we aimed to evaluate postoperative radiotherapy treatment patterns and the impact of timing of radiotherapy on survival outcomes in patients with glioblastoma using a large, national hospital-based registry, in the era of Stupp chemoradiation [8].

## MATERIALS AND METHODS

### Database and cohort selection

The National Cancer Database (NCDB) is a joint program of the Commission on Cancer (CoC) of the American College of Surgeons and the American Cancer Society. It is a hospital-based registry that collects data from more than 1500 CoC-accredited cancer hospitals and contains detailed information, including demographics, disease stage, comorbidity, radiation, surgery, and chemotherapy delivered during the first course of treatment on 70% of all incident cancer cases in the USA. The American College of Surgeons and the CoC have not verified and are not responsible for the analytic or statistical methodology used or for the conclusions drawn from these data by the investigator. The following NCDB analysis was performed with the approval of our institutional review board.

We queried the NCDB 2014 Participant User File for adult patients with primary glioblastoma using the International Classification of Diseases for Oncology histology code 9440 and site-specific codes C720–3, and included only patients with WHO Grade IV disease. We included patients diagnosed between 2010 and 2013 to ensure adequate follow-up and vital status information, and because site-specific factors such as extent of resection were consistently available beginning in 2010. We included patients who received radiation and chemotherapy within 60 days of surgery to minimize the inclusion of patients who received treatment for progressive disease. To address immortal time bias, we excluded patients who died or were lost to follow-up during the 60 day time interval after surgery. We required patients to have a known date of definitive surgery or biopsy and radiation start (See Table 1 for cohort selection).

**Table 1.** Cohort selection

	No.	%
Patients with primary glioblastoma diagnosed 2010–2013	38 694	100.0%
Exclude patients where diagnosis dates precedes reference dates to ensure data completeness	38 515	99.5%
Limit to patients where glioblastoma diagnosis is first cancer diagnosis	33 430	86.4%
Limit to patients 18 years or older	33 092	85.5%
Required to have known vital status	33 092	85.5%
All patients need to have known date of definitive surgery or biopsy; exclude patients who were diagnosed at autopsy or with extent of resection unknown	22 313	57.7%
All patients need to have both radiation and chemotherapy within 60 days of surgery	15 522	40.1%
Restrict to patients who received adjuvant chemotherapy	13 048	33.7%
Exclude patients who died or lost follow-up within 60 days of surgery	12 859	33.2%
Exclude patients where cases diagnosed at reporting facility did not receive any treatment at that facility	12 738	32.9%

### Covariates

We included relevant patient, tumor and treatment characteristics from the database in our analysis. Patient comorbidities were categorized as 0, 1 or 2 according to the Charlson–Deyo comorbidity score [9]. Distance from facility was based on the ‘great circle’ distance in miles between the patient’s residence and the hospital that reported the case, and was dichotomized into categories of ≤50 miles and >50 miles [10]. Residence (metropolitan, urban, or rural) was coded according to published files by the US Department of Agriculture Economic Research Service. Median household income for each patient’s zip code of residence was derived from 2012 US Census data. Reporting facilities were assigned a category classification by the CoC program. Time from definitive surgery or biopsy to radiation initiation was categorized into quartiles.

### Statistical analysis

Pearson chi-square tests were used to assess associations between baseline characteristics and age groups. Our primary outcome was overall survival. Survival time was defined as time from definitive surgery or biopsy to date of death or last follow-up. We assessed the relation between time from surgery to radiation initiation and all-cause mortality using Cox proportional hazard modeling. We included in our adjusted models relevant patient, tumor and treatment covariates (age, gender, race, comorbidity score, year of diagnosis, patient distance from reporting facility, zip code income, radiation dose, insurance type, and urban/metro/rural residence) that were selected *a priori* based on clinical judgment. In order to identify the optimal time from surgery/biopsy to radiation start, we classified time into the following categories: <15 days, 15–21 days, 22–28 days, 29–35 days, 36–42 days and >42 days.

Because facility type and region of the country are suppressed in the NCDB for patients younger than 40 years, we did not include these variables in our main multivariate models. Instead, we stratified our models on facility as there may be different institutional practices with regard to starting radiation. We performed sensitivity analyses in which we additionally adjusted for facility type and region of the country.

We used multivariate logistic modeling to determine factors independently associated with receipt of delayed radiation treatment. We initially included the same patient and tumor characteristics as above, and used a reverse, stepwise selection process to construct a working model, retaining variables with *P* < 0.1.

All tests were 2-sided, and *P* < 0.05 was considered statistically significant. Statistical analyses were performed using SAS Enterprise Guide (version 7.12, SAS Institute, Cary, North Carolina).

## RESULTS

### Patient and treatment characteristics

**Fig. 1.**
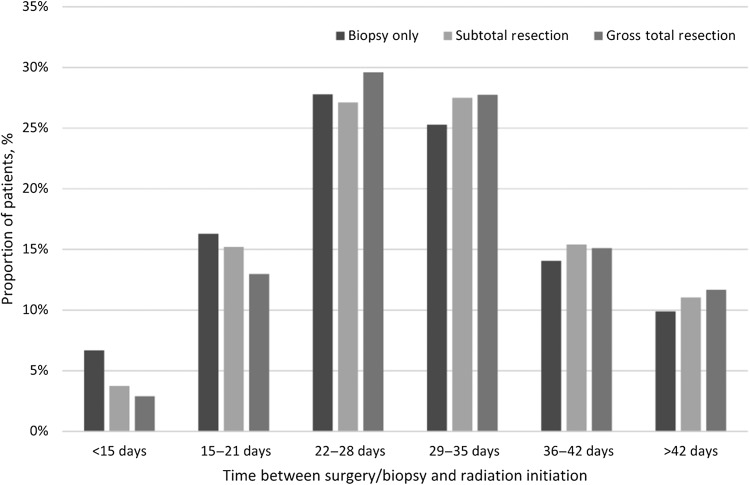
Distribution of time to radiation initiation from date of biopsy/surgery by extent of resection in entire study cohort.

Table 1 shows that a total of 12 738 patients met our inclusion criteria after our cohort selection process. The majority of patients underwent either gross total (*n* = 5270, 41%) or subtotal (*n* = 4700, 37%) resection, while 2768 patients (22%) underwent biopsy only. The majority of patients received 60 Gy or higher of adjuvant radiation (*n* = 8461, 66%). The median time from definitive surgery or biopsy and initiation of radiation was 29 days (interquartile range 24–36 days). Figure 1 shows the distribution of time to radiation initiation by extent of resection in our entire cohort.

Table 2 shows baseline patient and treatment characteristics by extent of resection for our entire cohort. Patients who resided in lower income areas, had Medicare/government insurance or no insurance, and had higher comorbidity scores were less likely to undergo gross total resection. White patients and patients treated at integrated network cancer programs were more likely to undergo gross total resection.

**Table 2.** Baseline patient and treatment characteristics by extent of surgery

Total patients	Biopsy only		Subtotal resection		Gross total resection		*P* value^a^
	2768		4700		5270		
Age							0.33
50 years or younger	536	(19.4%)	900	(19.1%)	1046	(19.8%)	
51–60 years	791	(28.6%)	1434	(30.5%)	1582	(30.0%)	
61–69 years	861	(31.1%)	1453	(30.9%)	1565	(29.7%)	
70 years or older	580	(21.0%)	913	(19.4%)	1077	(20.4%)	
Gender							0.05
Male	1599	(57.8%)	2839	(60.4%)	3085	(58.5%)	
Female	1169	(42.2%)	1861	(39.6%)	2185	(41.5%)	
Race							0.003
White	2532	(91.5%)	4236	(90.1%)	4866	(92.3%)	
Black	138	(5.0%)	274	(5.8%)	228	(4.3%)	
Other	98	(3.5%)	190	(4.0%)	176	(3.3%)	
Year of diagnosis							<0.0001
2010	641	(23.2%)	1111	(23.6%)	1086	(20.6%)	
2011	719	(26.0%)	1143	(24.3%)	1217	(23.1%)	
2012	677	(24.5%)	1195	(25.4%)	1430	(27.1%)	
2013	731	(26.4%)	1251	(26.6%)	1537	(29.2%)	
Comorbidity score							0.0006
0	2039	(73.7%)	3460	(73.6%)	4030	(76.5%)	
1	452	(16.3%)	778	(16.6%)	830	(15.7%)	
2 or higher	277	(10.0%)	462	(9.8%)	410	(7.8%)	
Location^b^							<0.0001
Northeast	690	(24.9%)	983	(20.9%)	1060	(20.1%)	
South	850	(30.7%)	1448	(30.8%)	1578	(29.9%)	
Central	661	(23.9%)	1179	(25.1%)	1354	(25.7%)	
West	420	(15.2%)	862	(18.3%)	976	(18.5%)	
Unknown	147	(5.3%)	228	(4.9%)	302	(5.7%)	
Facility type^b^							0.002
Community cancer program	100	(3.6%)	221	(4.7%)	206	(3.9%)	
Comprehensive community cancer program	958	(34.6%)	1586	(33.7%)	1754	(33.3%)	
Academic/Research program	1286	(46.5%)	2187	(46.5%)	2365	(44.9%)	
Integrated network cancer program	277	(10.0%)	478	(10.2%)	643	(12.2%)	
Other/Unknown	147	(5.3%)	228	(4.9%)	302	(5.7%)	
Distance from reporting facility							0.04
≤50 miles	2360	(85.3%)	3952	(84.1%)	4498	(85.4%)	
>50 miles	369	(13.3%)	702	(14.9%)	728	(13.8%)	
Unknown	39	(1.4%)	46	(1.0%)	44	(0.8%)	
Residence							0.42
Metropolitan	2226	(80.4%)	3731	(79.4%)	4265	(80.9%)	
Urban/Rural	448	(16.2%)	803	(17.1%)	828	(15.7%)	
Unknown	94	(3.4%)	166	(3.5%)	177	(3.4%)	
Income^c^							0.02
<$48 000	979	(35.4%)	1590	(33.8%)	1715	(32.5%)	
$48 000 or higher	1751	(63.3%)	3064	(65.2%)	3509	(66.6%)	
Unknown	38	(1.4%)	46	(1.0%)	46	(0.9%)	
Insurance							0.009
Medicaid/Not insured/Other gov	356	(12.9%)	617	(13.1%)	573	(10.9%)	
Private/Managed care	1441	(52.1%)	2507	(53.3%)	2910	(55.2%)	
Medicare	940	(34.0%)	1525	(32.4%)	1732	(32.9%)	
Unknown	31	(1.1%)	51	(1.1%)	55	(1.0%)	
RT dose							<0.0001
<60 Gy	797	(28.8%)	1346	(28.6%)	1248	(23.7%)	
60 Gy or higher	1772	(64.0%)	3063	(65.2%)	3626	(68.8%)	
Unknown	199	(7.2%)	291	(6.2%)	396	(7.5%)	

^a^Chi-square *P*-value.

^b^Suppressed for those under the age of 40.

^c^Median household income for each patient’s zip code of residence derived from year 2012 US Census data.

### Association between time to radiation initiation and survival

The median survival times (conditional on having survived 2 months as required for inclusion in this study) for those who had biopsy only, subtotal resection, and gross total resection were 14.2 months [95% confidence interval (CI) 13.6–14.8], 14.3 months (95% CI 14.0–14.7) and 17.8 months (95% CI 17.4–18.3), respectively (log-rank *P* < 0.0001).

We examined the impact of time to radiation initiation and survival, stratified by extent of surgery due to a significant interaction (*P* < 0.0001) between extent of surgery and time to radiation initiation that we found in our survival models. Table 3 shows the results of our adjusted models.

**Table 3.** Association between various cut-points for time from surgery/biopsy and radiation start and survival

	Biopsy only			Subtotal resection			Gross total resection		
	Adjusted hazard ratio^a^	95% CI	*P*	Adjusted hazard ratio^a^	95% CI	*P*	Adjusted hazard ratio^a^	95% CI	*P*
<15 days	1.67	1.24–2.25	0.0007	1.12	0.86–1.46	0.39	1.17	0.88–1.56	0.29
15–21 days	1.11	0.88–1.40	0.39	1.11	0.93–1.13	0.26	0.82	0.69–0.98	0.03
22–28 days	0.96	0.77–1.19	0.69	0.94	0.80–1.09	0.40	0.89	0.77–1.03	0.12
29–35 days	0.94	0.76–1.17	0.58	0.94	0.80–1.09	0.40	0.88	0.76–1.01	0.07
36–42 days	0.94	0.74–1.19	0.59	0.95	0.81–1.13	0.58	0.97	0.83–1.14	0.71
>42 days	Reference			Reference			Reference		

^a^Adjusted by age, gender, race, comorbidity score, year of diagnosis, patient distance from reporting facility, zip code income, radiation dose, insurance type, and urban/metro/rural residence, and stratified by facility.

For patients who had biopsy or subtotal resection, earlier initiation of radiation did not appear to be associated with improved survival. In fact, among patients who underwent biopsy only, there was significantly worse survival (hazard ratio 1.67, 95% CI 1.24–2.25, *P* = 0.0007) with earlier initiation of radiation (within 15 days of biopsy) compared with delayed radiation (defined as >42 days after surgery or biopsy). However, among patients who underwent gross total resection, there appeared to be improved survival with early initiation of radiation. Patients who initiated radiation within 15–21 days of gross total resection had improved survival (hazard ratio 0.82, 95% CI 0.69–0.98, *P* = 0.03) compared with patients who had delayed radiation. There was also a trend (*P* = 0.07–0.12) for improved survival for patients who initiated radiation within 22–35 days of gross total resection compared with patients who had delayed radiation.

### Factors associated with delayed radiation after gross total resection

Using 35 days as a cut-off based on our Table 3 results, we then looked at factors that were independently associated with receiving radiation >35 days after gross total resection (Table 4). Patients who were black, had Medicaid or other government insurance or were not insured, and who lived in metropolitan areas or further away from the treating facility had higher odds of receiving radiation >35 days after gross total resection. Patients who lived in higher income areas had lower odds of receiving radiation >35 days after gross total resection.

**Table 4.** Factors associated with radiation initiation >35 days after surgery among patients with gross total resection

	Adjusted odds ratio	95% CI	*P*
Race			0.06
White	Reference		
Black	1.41	1.05–1.89	
Other	1.14	0.81–1.59	
Residence			0.003
Urban/Rural	Reference		
Metropolitan	1.36	1.11–1.66	
Distance from reporting facility			0.05
≤50 miles	Reference		
>50 miles	1.22	1.00–1.48	
Insurance			0.001
Medicare	Reference		
Medicaid/Not insured/Other government	1.42	1.15–1.75	
Private/Managed care	0.98	0.86–1.13	
Income			0.03
<$48 000	Reference		
$48 000 or higher	0.85	0.74–0.98	

Variables with *P* < 0.1 were retained in the multivariable logistic model.

### Sensitivity analyses

We performed sensitivity analyses adjusting for facility type and region of the country (available for patients 40 years and older), and found that our results were materially unchanged. Patients who initiated radiation within 15–21 days of gross total resection had improved survival (hazard ratio 0.80, 95% CI 0.66–0.95, *P* = 0.01) compared with patients who had delayed radiation. There continued to be a trend for improved survival for patients who initiated radiation within 22–35 days of gross total resection compared with patients who initiated radiation after 35 days of gross total resection (data not shown).

## DISCUSSION

Using a large, nationally representative sample of patients with glioblastoma treated with chemotherapy and radiation, we found that there may be a survival benefit with earlier initiation of radiation after gross total resection. However, there appeared to be no such association for patients who received subtotal resection and biopsy only, and even possible survival detriment associated with early initiation of radiation after biopsy only.

Others have reported findings of increased survival with earlier initiation of radiation treatment for patients with Grade III or IV gliomas in the pre-temozolomide era. Do *et al.* found a 2% increased risk of death for each additional day after presentation before initiation of radiation [4]. Irwin *et al.* similarly reported a 9% increased risk of death per week of delay [5]. More modern series of patients with glioblastoma treated with both adjuvant chemotherapy and radiotherapy found that starting radiation within 42 days of surgery was associated with improved progression-free and/or overall survival [8, 11, 12]. Corroborating our data, Valduvieco *et al.* analyzed only patients who had gross total resection and found improved survival with earlier initiation of radiation [13]. Given the short doubling time of GBM [14], it is intuitive that timely access to radiation treatment may result in improved outcomes.

However, multiple other studies have shown that time to radiation start has no association with survival outcomes [3, 6, 7, 15]. Blumenthal *et al.* included thousands of GBM patients treated on Radiation Therapy Oncology Group (RTOG) trials in their analysis, and found significantly better, rather than inferior, survival with a wait time to radiation start of more than 4 weeks (but less than 6 weeks) [3]. There are several possible explanations for the unexpected finding of inferior outcomes with earlier initiation of radiation. Experimental animal data suggests that early delivery of radiation after surgery may result in higher levels of brain tissue damage [16]. Postsurgical hypoxia and edema in the immediate postoperative period may induce radioresistance [17], resulting in radiation treatment being less effective. Additionally, as the surgical cavity shrinks over time after surgery, irradiating too early could result in a larger irradiated volume and lead to more toxicity. Finally, selection bias may explain these retrospective findings of survival detriment of early initiation of radiation: patients with the most adverse characteristics are likely the ones who received radiation sooner. For example, Blumenthal found that those receiving radiation earlier were more likely to have a poor performance status, have worse neurologic impairment, have undergone biopsy only, and have a worse recursive portioning analysis (RPA) class [3].

Thus, we analyzed the time interval between surgery/biopsy and radiation initiation in finer intervals to tease out the various confounders that may be at play. In doing so, we found a trend to inferior survival with early initiation of radiation (within 15 days of surgery) among patients who underwent biopsy only, and believe that this may be due to selection bias as described above. Additionally, patients who received biopsy had worse survival than patients who underwent gross total resection, and they may not have lived long enough to receive the full benefit of early radiation initiation. While it also appeared that among those who had gross total resection, those who initiated radiation within 15 days of surgery did not have improved survival compared with those who had delayed radiation, those patients who initiated radiation within 15 to 35 days of surgery did have a survival benefit. Furthermore, earlier initiation of radiation was associated with improved outcomes, with patients who received radiation within 15–21 days of surgery having the lowest hazard of death compared with those who initiated radiation more than 42 days after surgery. Initiating radiation too early (within 2 weeks of surgery) may not be optimal due to the need for post-operative healing, although there may also be a component of selection bias behind these results.

Interestingly, we found that there are various socio-economic factors that are associated with timely initiation of radiation. Although we cannot deduce the reasons for radiation delay from the NCDB, we found that patients who were black and underinsured, and who lived in metropolitan areas or further away from the treating facility were more likely to have delayed radiation, while patients who lived in higher income zip codes were less likely to receive delayed radiation. We also found similar socio-demographic associations with extent of surgery that patients received. Other have also found similar associations between glioblastoma treatment and outcomes and socioeconomic factors such as race and insurance status [18–20].

Limitations of our study include its retrospective nature, lack of correction for multiple testing and lack of important covariates. For example, Karnofsky Performance Status (KPS) and molecular data such as MGMT methylation status were missing for the majority of patients in our cohort. Although we controlled for a large number of observed covariates, there remains the possibility of selection bias and unobserved confounding. However, this is a question that is unlikely to be addressed in the randomized setting. We attempted to address the confounder of performance status by limiting our cohort to those who were robust enough to receive both chemotherapy and radiation within 2 months of surgery. Additionally, NCDB lacks data on tumor progression status. However, because GBM is a highly fatal disease, we expect overall survival to closely mirror progression-free survival.

## CONCLUSIONS

In a large cohort of patients with glioblastoma treated with chemoradiation across the country, we found that there may be a survival benefit by initiating radiotherapy within the 35 days following gross total resection. Further work is warranted in understanding barriers to timely access to optimal therapy.

## CONFLICT OF INTEREST

Scott G. Soltys is a consultant for Inovio Pharmaceuticals, Inc.

There are no conflicts of interest to disclose.

## FUNDING

KL2 Mentored Career Development Award of the Stanford Clinical and Translational Science Award to Spectrum (NIH KL2 TR 001083)—Erqi Pollom.
